# Biosynthesis, characterization and optimization of TiO_2_ nanoparticles by novel marine halophilic *Halomonas* sp. RAM2: application of natural dye-sensitized solar cells

**DOI:** 10.1186/s12934-023-02093-3

**Published:** 2023-04-21

**Authors:** Rasha A. Metwally, Jehan El Nady, Shaker Ebrahim, Amany El Sikaily, Nermeen A. El-Sersy, Soraya A. Sabry, Hanan A. Ghozlan

**Affiliations:** 1 Marine Microbiology Lab., National Institute of Oceanography and Fisheries, NIOF, Alexandria, Egypt; 2 Electronic Materials Department, Advanced Technology and New Materials Research Institute, City of Scientific Research and Technological Applications (SRTA-City), New Borg El-Arab City, Alexandria Egypt; 3 Department of Materials Science, Institute of Graduate Studies and Research, Alexandria University, Alexandria, Egypt; 4 Marine Pollution Lab., National Institute of Oceanography and Fisheries, NIOF, Alexandria, Egypt; 5 Botany & Microbiology Department, Faculty of Science, Alexandria University, Alexandria, Egypt

**Keywords:** TiO_2_ nanoparticles, Carotenoids, Dye-sensitized solar cells, *Halomonas*, *Kocuria*, Bacterioruberins, Spirilloxanthin

## Abstract

**Background:**

Metal oxide nanoparticles (NPs) are becoming valuable due to their novel applications. The green synthesis of TiO_2_ NPs is more popular as a flexible and eco-friendly method compared to traditional chemical synthesis methods. TiO_2_ NPs are the most commonly used semiconductor in dye-sensitized solar cells (DSSCs).

**Results:**

The biogenic TiO_2_ NPs were produced extracellularly by the marine halophilic bacterium *Halomonas* sp. RAM2. Response surface methodology (RSM) was used to optimize the biosynthesis process, resulting in a starting TiO_2_ concentration of 0.031 M and a pH of 5 for 92 min (⁓15 nm). TiO_2_ NPs were well-characterized after the calcination process at different temperatures of 500, 600, 700 and 800 °C. Anatase TiO_2_ NPs (calcined at 500 °C) with a smaller surface area and a wider bandgap were nominated for use in natural dye-sensitized solar cells (NDSSCs). The natural dye used as a photosensitizer is a mixture of three carotenoids extracted from the marine bacterium *Kocuria* sp. RAM1. NDSSCs were evaluated under standard illumination. After optimization of the counter electrode, NDSSC_Bio(10)_ (10 layers) demonstrated the highest photoelectric conversion efficiency (η) of 0.44%, which was almost as good as NDSSC_P25_ (0.55%).

**Conclusion:**

The obtained results confirmed the successful green synthesis of TiO_2_ NPs and suggested a novel use in combination with bacterial carotenoids in DSSC fabrication, which represents an initial step for further efficiency enhancement studies.

## Background

Nanotechnology has profoundly transformed science and plays a worthy role in several innovative aspects of the new millennium. Particularly in comparison to bulk materials, NPs have distinct physical, chemical, and biological properties. It is widely assumed that the basic properties of nanostructured materials are mediated by their sizes and shapes. As a consequence, hard investigations have been made to control the appropriate morphologies of these nanostructure materials [1].

Metal oxide nanoparticles constitute one of the most multi-functional and ubiquitously used types, with applications in electronics, chemistry, catalysts, and medical and pharmaceutical disciplines [2]. Among them are titanium dioxide nanoparticles (TiO_2_ NPs), which have become a great addition to nanotechnology due to their tremendous applications as photocatalysts and UV absorbers [3, 4].

Chemical, physical, and biological processes can all be used to construct crystalline TiO_2_ nanoparticles with distinct morphologies [5]. Regrettably, toxic chemicals and time-consuming procedures in traditional synthesis techniques frequently endanger humans and the environment. Biosynthesized nanoparticles are eco-friendly and safe, and have been incorporated into various successful and efficient applications as reported in many studies. The biosynthesis of TiO_2_ NPs using microorganisms is an alternative green route to overcome these disadvantages while maintaining their excellent properties [6]. During the biological synthesis of NPs, microbial metabolites including enzymes, terpenoids, and phenolics act as stabilizers and capping agents [7]. A major field of interest is the advantage of flexibility in monitoring the experimental conditions of nanoparticle microbial synthesis, such as pH and temperature, which influence the physicochemical characteristics such as morphology, stability, and properties of the biosynthesized nanoparticles [8].

Although green synthesis of nanoparticles is a part of bioinspired protocols, several challenges should be considered. Material availability, selection, collection, reaction conditions, quality management and application face challenges for large-scale applications in industry [9]. Both the size and shape of NPs are highly influenced by the prepared biological extracts. As a result, it is critical to find the ideal conditions and components in order to implement and optimize the synthesis protocol for the purpose of getting NPs with the required size, shape, and surface charges [10]. Green and nontoxic reducing agents were an important question in the green synthesis of NPs, as they are weak to form high-quality NPs. Thus, researchers seek to find stronger green reducing agents or optimum reaction conditions that support the formation of the desired high-quality NPs; this continues to be a tricky and critical challenge [11]. NPs face characterization challenges, which have a major impact on the accuracy of the detailed characterization, as deciding on an appropriate characterization technique is thus critical [12].

Analytical techniques such as transmission electron microscopy (TEM), selected area electron diffraction (SAED), energy dispersive X-ray (EDX), X-ray diffraction (XRD), UV–visible spectroscopy, and Brunauer-Emmett and Teller (BET) surface area can be used to explore important characters such as the size, phase, surface area and band gap of the synthesized TiO_2_ NPs [13].

Recently, researchers concluded that marine bacteria, particularly the halophilic ones, are valuable and unique sources of bioactive compounds and have enzymatic activities with properties distinct from those of conventional enzymes [14]. So, in this study, the green TiO_2_ NPs synthesis was selected by using the marine halophilic bacteria *Halomonas* sp. RAM2 via the extracellular route.

Solar energy offers an environmentally friendly alternative to meet the world's growing energy demand. Hence, photovoltaic devices that help in the conversion of solar energy into electricity have gained a great deal of attention recently [15]. DSSCs have sparked impressive attention for their structure simplicity, relatively low cost, and encouraging efficiency in transforming solar energy into electricity [16]. O'Regan and Grätzel pioneered this technology in 1991 [17].

TiO_2_ is an important photocatalytic material in DSSCs that exists in two main phases: anatase and rutile [18]. Although rutile seems to be the most thermodynamically stable phase, anatase is chosen due to its larger band gap for DSSCs [19]. TiO_2_ is commonly used as a semiconducting layer due to its non-toxicity, low cost, and wide availability [20]. Improving functionalities in solar cells are influenced by the size of nanocrystals during a solid–solid phase transition. So, phase control is a critical step [21].

In general, DSSCs are assembled from the photoanode (working electrode), which is mainly a conductive transparent substrate (fluorine-doped tin oxide (FTO) glass), with the use of an overlying semiconductor film such as TiO_2_ that adsorbs the photosensitizer dye, providing the photoelectrons [22]. The working electrode is coupled with the counter electrode that serves as a reduction catalyst. In between, an electrolyte that functions as a redox couple is injected [20, 23].

Photosensitizers are among the most key parts of DSSC and have been extensively studied in the last 20 years, with thousands of dyes suggested and evaluated for this type of application [24]. Till now, DSSCs have been based on a single sensitizer such as ruthenium or porphyrin dyes, which have some limitations such as rareness, purification difficulties and environmental hazards [25]. So, renewable, eco-friendly, and non-carcinogenic natural dyes have attracted attention as photosensitizers [26].

One of the natural pigments is carotenoids, which come in various colors, ranging from yellow to orange and red. Over 750 carotenoids have been observed in plants, fungi, and microorganisms with a wide range of significant biological functions, including light-harvesting, photoprotection and antioxidants [27, 28]. They are classified as C_30_, C_40_, C_42_ or C_50_ based on the number of carbons in their carotene backbones [29]. As an example of the uncommon carotenoids used in the present study, those that were extracted from the marine bacterium *Kocuria* sp. RAM1. *Kocuria* sp. RAM1 dye is a mixture of three carotenoid compounds, namely bisanhydrobacterioruberin, trisanhydrobacterioruberin (C_50_-carotenoids) and 3,4,3',4'-tetrahydrospirilloxanthin (C_42_-carotenoids) [30].

The counter electrode plays a role in collecting and transferring electrons from the external circuit and regenerates the dye by catalyzing electrolyte reduction**.** Pt-coated FTO is widely used as a counter electrode, but there is an interest in replacing it due to its high cost [31]. One alternative to the Pt counter electrode is the Cu_2_S counter electrode, which has good performance for polysulfide electrolytes at a low cost. In addition, Cu_2_S counter electrodes can be simply prepared by the successive ionic layer adsorption and reaction (SILAR) technique, which controls the film microtopography through changing deposition times [32].

The basic component in DSSCs is the electrolyte, which is critical for the inner charge carrier transport between the two electrodes and regenerates the dye and itself continuously, thus significantly impacting the efficiency and stability of the systems [32, 33]. Several studies reported the use of various redox couples of electrolytes other than I^−^/I^3−^ to enhance the durability of DSSCs, such as the redox couple of polysulfide electrolytes (S^2−^/S_x_^2−^) [34].

As green nanoparticle synthesis remains a challenge, the present study investigated the synthesis and optimization of TiO_2_ NPs by newly isolated marine halophilic bacterium *Halomonas* sp. RAM2 and its application in fabricating a novel DSSC using a photosensitizer of carotenoids extracted from the marine bacteria *Kocuria* sp. RAM1. Cu_2_S counter electrode was prepared by the SILAR technique and optimized trying to enhance the efficiency, but further study is required to improve the overall conversion efficiency.

## Results

### Isolation, screening and molecular identification

**Fig. 1:**
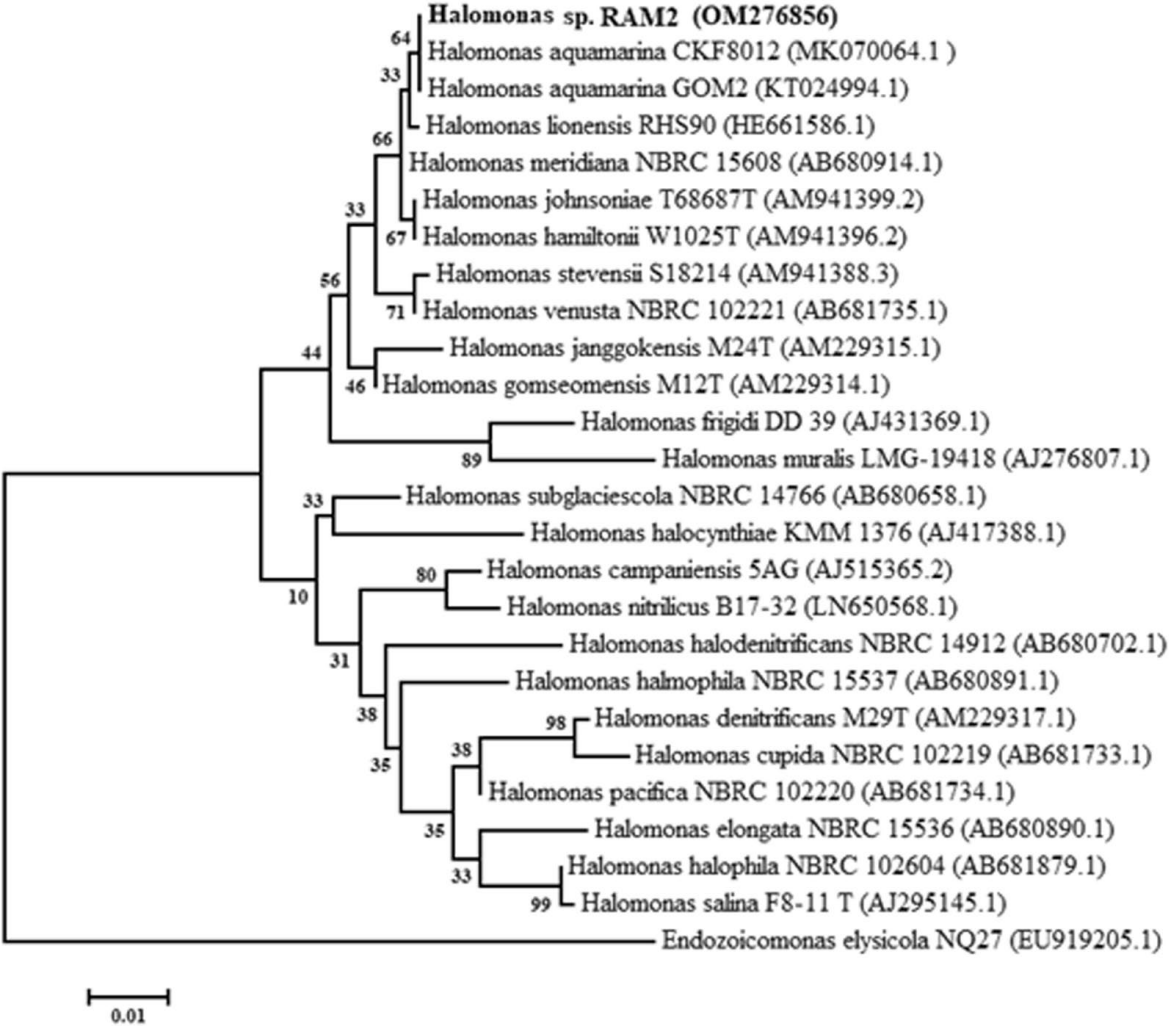
A phylogenetic tree of *Halomonas* sp. RAM2. The isolate is denoted by a bold style font. GenBank sequence accession numbers are shown in parenthesis after naming the strains

A salt-tolerant colony was isolated from the sea urchin (*Echinometra mathaei*)*,* collected from Safaga, Red Sea, Egypt. The desirable bacterial isolate was purified and grown on a nutrient agar medium (2% NaCl). Colonies were round, smooth, raised, convex, opaque, and off-white-colored. Microscopic examination revealed that the cells were Gram-negative, non-spore-forming rods. The isolate was referred to as *Halomonas* sp. RAM2 as an outcome of molecular analysis, and the sequence was submitted to GenBank (at the NCBI Nucleotide Database with accession number OM276856) (Fig. 1).

### *Halomonas* sp. RAM2 growth

**Fig. 2:**
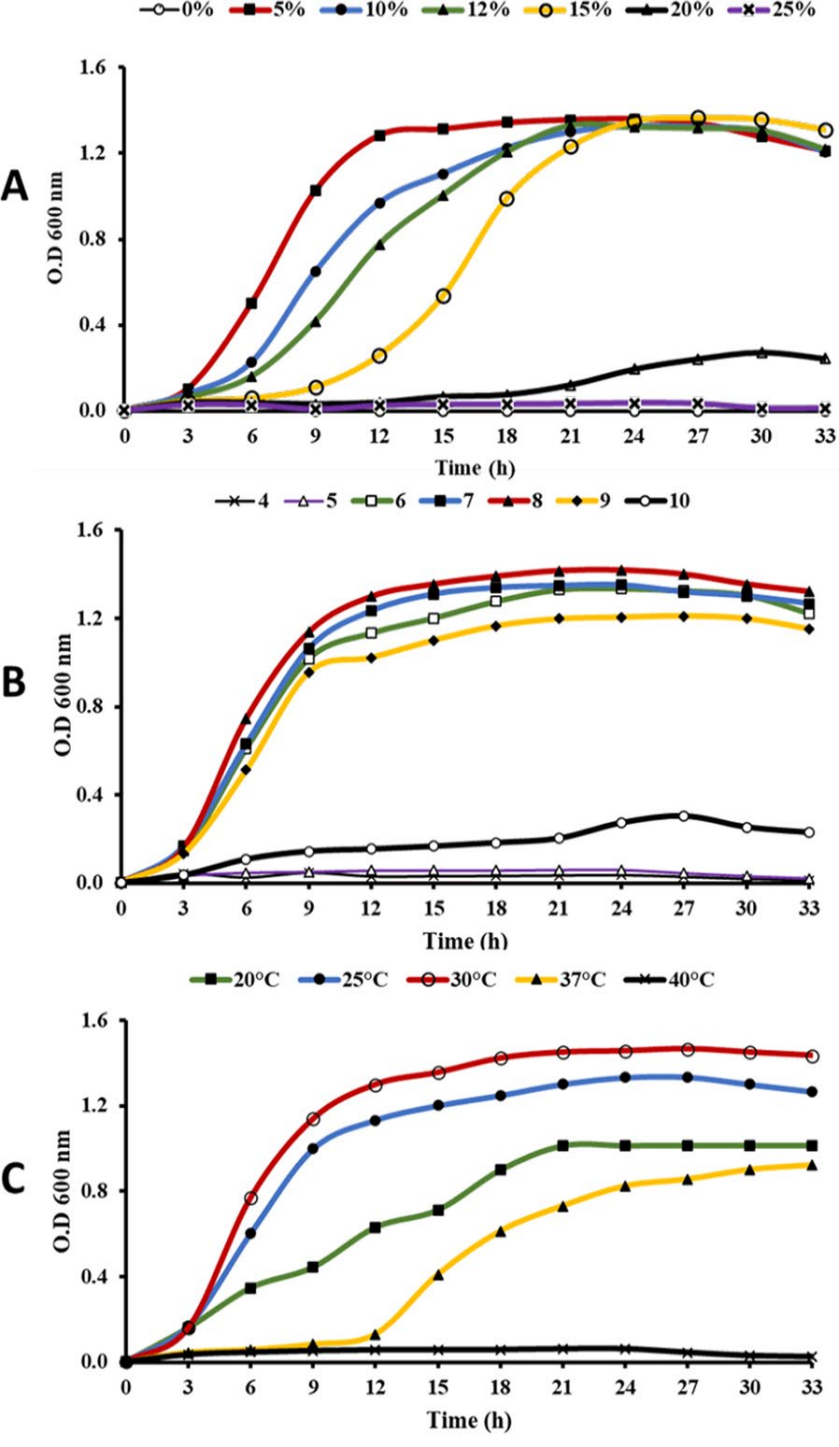
Growth of *Halomonas* sp. RAM2 in nutrient broth medium at **A** different NaCl concentrations at 30 °C and pH = 7; **B** different pH at 30 °C and 5% NaCl; and **C** different temperatures at pH = 8 and 5% NaCl

The physiological characterization of *Halomonas* sp. RAM2 was performed by analyzing salinity tolerance, pH and temperature (Fig. 2). The maximum growth of *Halomonas* sp. RAM2 was observed at 5% NaCl (O.D = 1.3) and it tolerated high salinity up to 15% after 33 h. No growth was observed in the absence of NaCl (Fig. 2A). Medium adjusted to pH 8 supported maximum growth (O.D = 1.4), whereas pH of 4, 5 and 10 recorded significant low growth (Fig. 2B). Bacterial growth was observed in a range of temperatures (20–37 °C), with maximum growth at 30 °C, while growth inhibition was observed at 40 °C (Fig. 2C).

### Biosynthesis and optimization conditions of TiO_2_ NPs

The current study deals with extracellular TiO_2_ NPs synthesis using cell-free filtrate of *Halomonas* sp. RAM2. The milky-colored suspension confirmed TiO_2_ NPs formation, followed by calcination at 500, 600, 700, and 800 °C for further characterization**.**

**Fig. 3:**
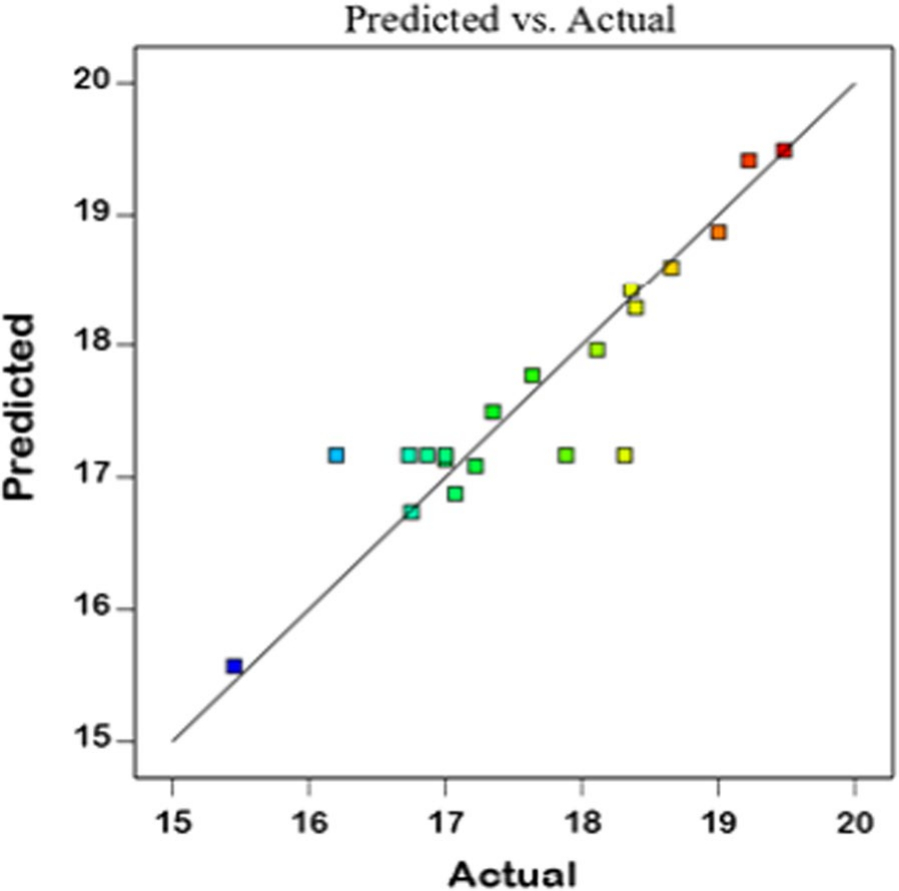
Actual and predicted plot of TiO_2_ NPs size (nm)

TiO_2_ concentration (A), pH (B) and reaction time (C) were considered for optimization using response surface methodology (RSM) across 20 runs (Table 1). It was observed that the average size of TiO_2_ NPs ranged from 15.45 to 19.48 nm. As per the analysis of variance (ANOVA), the model was statistically significant (*p*-value = 0.0129)**.** Moreover, there was no significant lack of fit for the model, thus suggesting that this model adequately fit the data. Additionally, the determination coefficient (R^2^ = 0.844) indicated that the model can explain 84.40% of the variation in the response, indicating the reliability of the model. The predicted R^2^ (0.7029) agreed reasonably well with the adjusted R^2^ (0.7036). The actual and predicted size averages of TiO_2_ NPs are shown in Fig. 3. The final practical model in terms of a coded factor (A = TiO_2_ concentration, B = pH, C = time) could be expressed as follows:

1 $${\text{TiO}}_{{2}} {\text{NPs size }}\left( {{\text{nm}}} \right) \, = { 17}.{17 } - \, 0.0{\text{535 A }} + \, 0.{8}0{\text{59 B }} - \, 0.{\text{5174 C }} + \, 0.0{\text{831 AB }} + \, 0.0{\text{672 AC }} - \, 0.{\text{2767 BC }} + \, 0.{\text{4757 A}}^{{2}} - \, 0.0{\text{847 B}}^{{2}} + \, 0.{\text{2962 C}}^{{2}}$$

**Table 1:** Experiment design of RSM for 3 operating independent variables affecting TiO_2_ NPs size

Run	Reaction conditions			Response	
	A	B	C	TiO_2_ Size (nm)	
	Conc. (M)	pH	Time (min)	Observed	Predicted
1	0.028	7	90	16.73	17.17
2	0.028	7	90	18.31	17.17
3	0.014	8.2	72	19.48	19.49
4	0.028	7	90	17.88	17.17
5	0.028	7	90	16.87	17.17
6	0.014	8.2	108	17.64	17.77
7	0.028	9	90	18.39	18.28
8	0.028	5	90	15.45	15.57
9	0.005	7	90	18.66	18.60
10	0.041	5.8	108	16.75	16.74
11	0.028	7	120	17.00	17.13
12	0.028	7	60	19.00	18.87
13	0.041	8.2	72	19.22	19.42
14	0.041	8.2	108	18.11	17.96
15	0.041	5.8	72	17.22	17.08
16	0.028	7	90	17.00	17.17
17	0.050	7	90	18.36	18.42
18	0.014	5.8	72	17.35	17.49
19	0.028	7	90	16.20	17.17
20	0.014	5.8	108	17.07	16.88

**Fig. 4:**
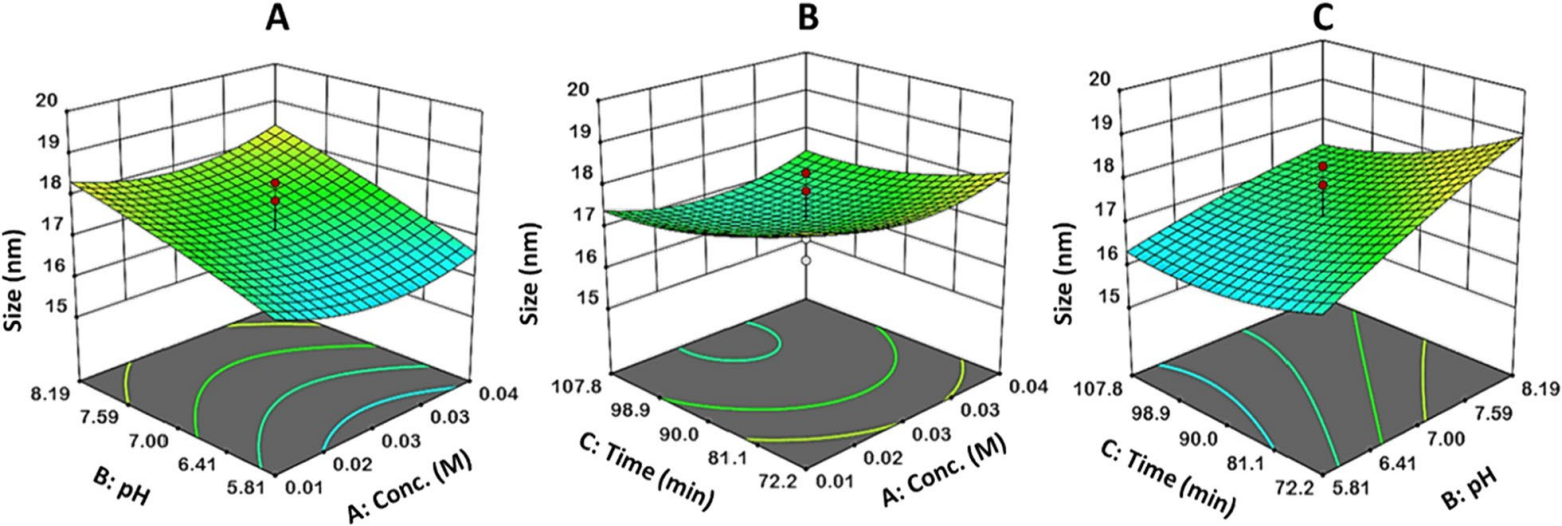
The 3D surface plots of the interaction effects on TiO_2_ NPs size between **A** concentration and pH, **B** concentration and time, and **C** pH and time

The interaction effect of the three factors on TiO_2_ NPs size is demonstrated in Fig. 4. The size of TiO_2_ NPs decreased as pH and TiO_2_ concentration decreased (Fig. 4A). The smaller size of NPs was also led by the longer duration and lower TiO_2_ concentration (Fig. 4B). Thus, the optimal reaction conditions for the smaller TiO_2_ NPs size were in the pH range of 5.8–6 with a TiO_2_ starting concentration of 0.03 M for 80–102 min.

For reaction optimization investigation, the desirability function (DF) was employed. The response value (TiO_2_ NP size) was set to a minimum. pH was set to 5, and concentration and time were set within the range for maximum desirability. The starting TiO_2_ concentration was 0.031 M, at pH = 5 for 92 min. The highest obtained desirability (0.975) was achieved with 15.5 nm TiO_2_ NPs. The reaction was validated to confirm the model’s adequacy under these predicted optimum conditions, yielding an experimental value of 15.9 nm, which was close to the predicted size (15.5 nm). The comparison that has been made between the predictive and experimental results at the optimum levels indicates that the model has high validity.

### Characterization of biogenic TiO_2_ NPs

**Fig. 5:**
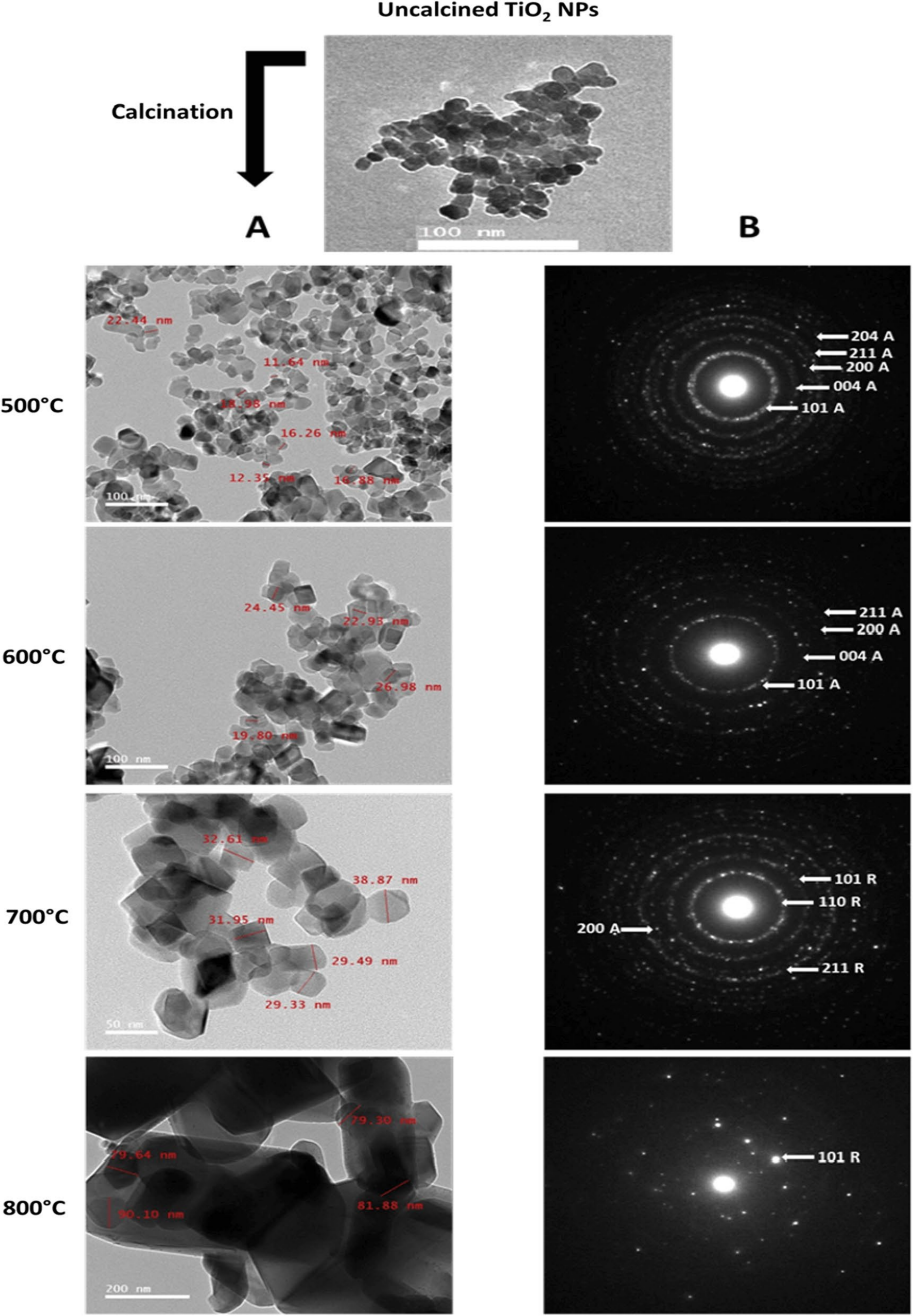
TEM micrographs of biosynthesized TiO_2_ NPs calcined at 500**,** 600**,** 700 and 800 °C **A** and the corresponding SAED patterns **B.** A = anatase, R = Rutile

*Transmission Electron Microscopy (TEM)* Micrographs clearly illustrate the well-defined cubic structure and the variation in size of the biosynthesized TiO_2_ NPs after calcination. The size range of samples calcined at 500, 600, 700 and 800 °C was 11–22, 19–26, 29–38 and > 80 nm, respectively (Fig. 5A). It is observed that the uncalcined TiO_2_ NPs coagulate while the calcined ones are dispersed uniformly.

*The selected area electron diffraction (SAED)* Patterns are displayed in Fig. 5B, which shows the crystalline nature of TiO_2_ NPs. The intense diffraction rings are indexed as the (101), (004), (200), (211) and (204) planes of the anatase TiO_2_, and the (101), (110) and (211) planes of the rutile TiO_2_.

**Fig. 6:**
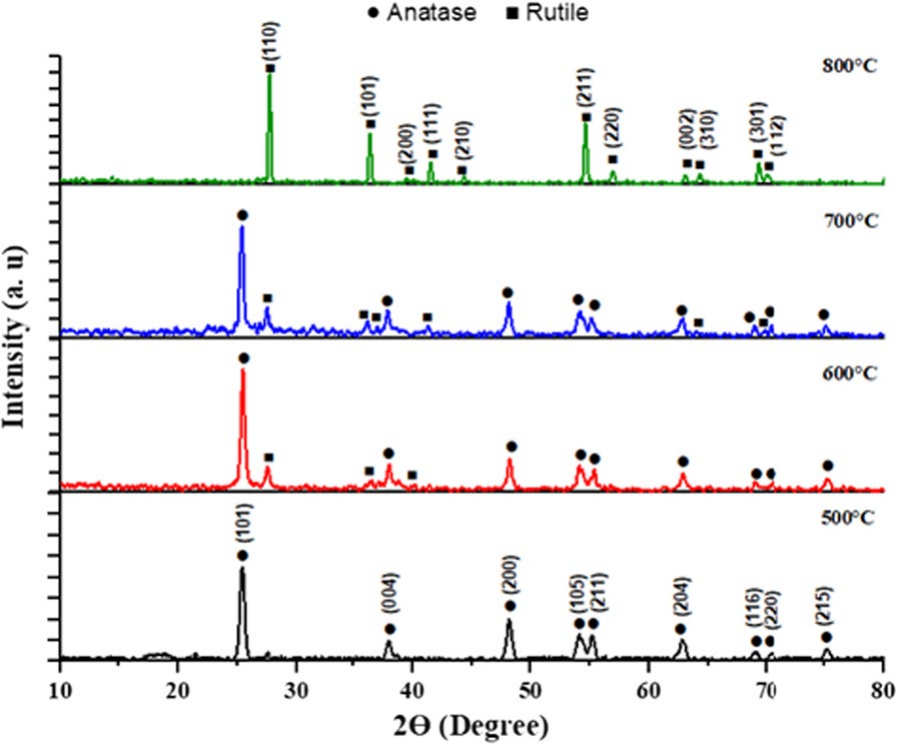
XRD patterns of biosynthesized TiO_2_ NPs calcined at 500, 600, 700 and 800 °C

*X-ray diffraction (XRD)* is a powerful technique for analyzing NPs crystallinity. A significant shift in the biosynthesized TiO_2_ NPs structure was observed after calcination (Fig. 6). Only the anatase TiO_2_ phase characteristic peaks (JCPDS 01-089-4921) were well-defined when the sample was calcined at 500 °C, indicating good crystallinity. The peaks indexed to the reflection from (101), (004), (200), (105), (211), (204), (116), (220) and (215) planes at 2θ values of 25.42°, 37.97°, 48.18°, 54.19°, 55.19°, 62.84°, 69.01°, 70.40° and 75.24° correspond to the anatase phase of TiO_2_, respectively. After calcination at 600 °C, new peaks corresponding to the rutile phase started to appear in a minor proportion. According to JCPDS 01-089-4920, the peaks at 27.68°, 36.32°, and 41.48° correspond to the (110), (101), and (111) of the rutile phase, respectively. At 700 °C, additional rutile phase peaks (310) and (301) appeared at 2θ of 64.32° and 69.24°, respectively. TiO_2_ NPs were completely transformed into the rutile phase with strong peaks at 800 °C, which were clearly represented in (110), (101), (200), (111), (210), (211), (220), (002), (310), (301) and (112) planes.

In terms of size, the temperature affected the size of TiO_2_ NPs. NPs calcined at 500 °C were found in the range of 10.16–19.08 nm. And as the temperature was raised to 600 °C, the size of the anatase phase increased from 13.65 up to 27.28 nm, while the new rutile crystal size ranged from 25 to 28.1 nm. At the anatase–rutile mixed phase of 700 °C, the rutile crystal average size was 30.29 nm, while some of the anatase crystal sizes started to reduce and a larger rutile crystal size was observed at 800 °C. As an outcome, increasing the temperature caused an anatase-to-rutile phase transition, which was characterized by an increase in TiO_2_ NPs crystal size.

**Fig. 7:**
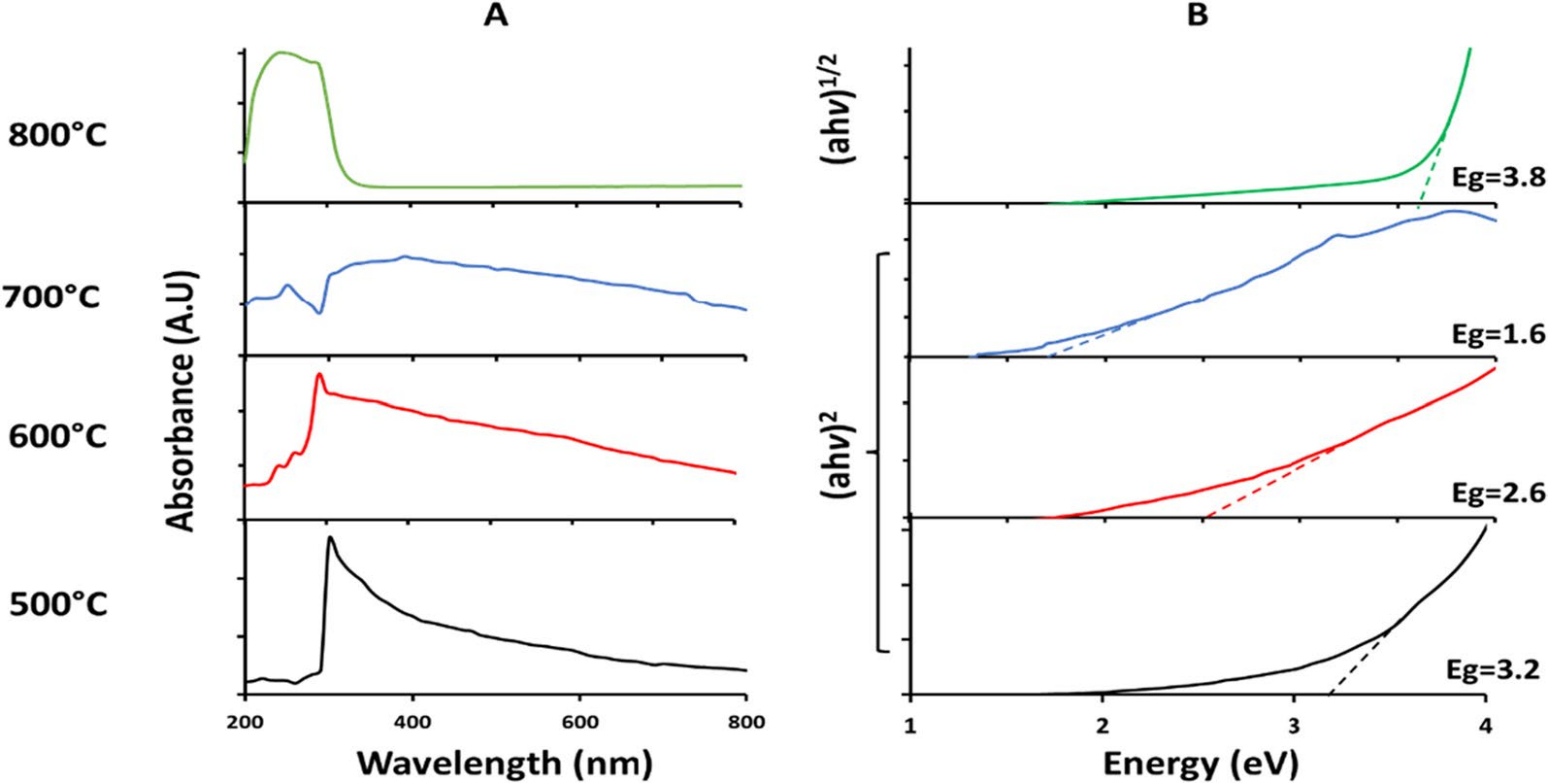
UV–Vis absorption **A** and the corresponding Tauc plots **B** of *Halomonas* sp. RAM2 TiO_2_ NPs calcined at 500, 600, 700 and 800 °C. An Indirect band gap was obtained for TiO_2_ NPs calcined at 500, 600 and 700 °C, while a direct band gap was obtained for TiO_2_ NPs calcined at 800 °C

*The optical properties* of TiO_2_ NPs were investigated in aqueous suspensions (Fig. 7A). Samples calcined at 500 and 600 °C showed almost similar absorption at 300 and 290 nm, respectively, indicating the beginning of the rutile phase with a small proportion. A higher absorption value of 390 nm was obtained at 700 °C, while the absorption of the rutile sample was observed at 250 nm.

The semiconductor band gaps of TiO_2_ NPs as determined via the Tauc plots are shown in Fig. 7B. The data were fitted to the indirect band gap for TiO_2_ NPs calcined at 500, 600, and 700 °C and were found to be 3.2, 2.6, and 1.6 eV, respectively. The decrease in the band gap energy with increasing calcination temperature indicates anatase to rutile phase transition, as proven by XRD analysis. TiO_2_ NPs calcined at 800 °C had a direct band gap of 3.8 eV, indicating a complete rutile phase.

**Fig. 8:**
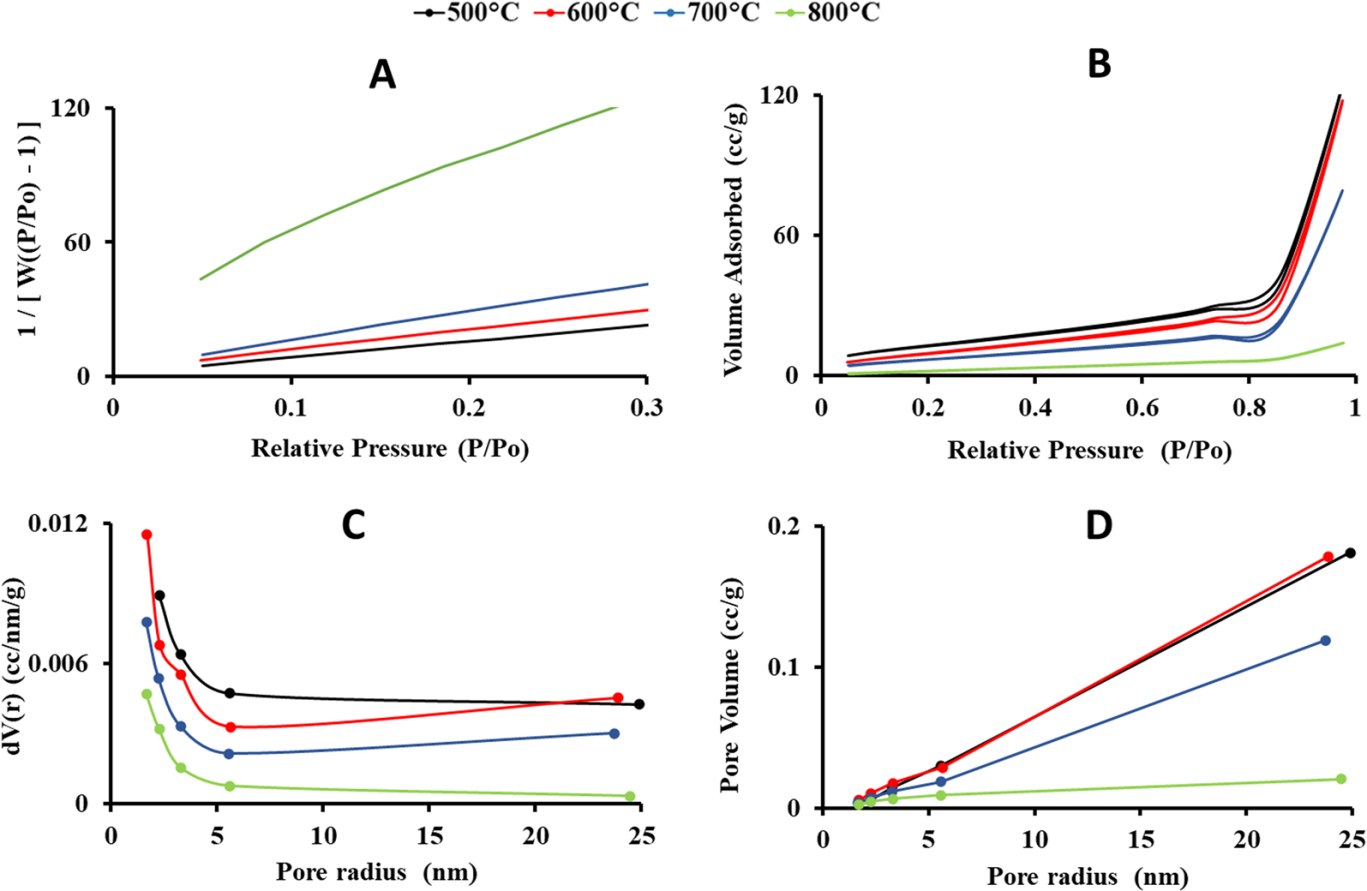
BET plots **A**, adsorption–desorption isotherms** B**, BJH pore size distribution **C** and cumulative pore volume **D** of *Halomonas* sp. RAM2 TiO_2_ NPs calcined at 500, 600, 700 and 800 °C

*The BET* surface area of samples calcined at 500, 600, and 700 °C was 47.96, 37.99, and 26.82 m^2^/g, respectively (Fig. 8A), while the surface area of the rutile TiO_2_ NPs was reduced significantly (9.99 m^2^/g), indicating the increase in NPs size with temperature. The nitrogen adsorption–desorption isotherm is shown in Fig. 8B. TiO_2_ NPs calcined at 500, 600, and 700 °C exhibited a characteristic type IV BET isotherm, indicating their porous nature, while the rutile TiO_2_ NPs exhibited a characteristic type III BET isotherm, which explains the lower surface area. The plot of dV (r) vs. pore radius (Fig. 8C) showed a distribution in pore size of 1–25 nm for all samples, with the high values around 1.6–2.2 nm. The concentrations display a decreasing trend with an increase in pore size in all samples**.** A sample calcined at 500 °C showed a higher number of pores with diameters of less than 3 nm compared to the other samples calcined at 600, 700, and 800 °C. The pore volumes were determined via the BJH model (Fig. 8D). Samples calcined at 500 and 600 °C exhibited the highest pore volume with slightly similar values (0.18 and 0.17 cc/g, respectively) and an average pore size of 2.28 and 1.68 nm, respectively. A sample calcined at 700 °C exhibited a pore volume of 0.12 cc/g and an average pore size of 1.68 nm, while the rutile TiO_2_ NPs exhibited a pore volume of 0.02 cc/g and an average pore size of 1.68 nm.

**Fig. 9:**
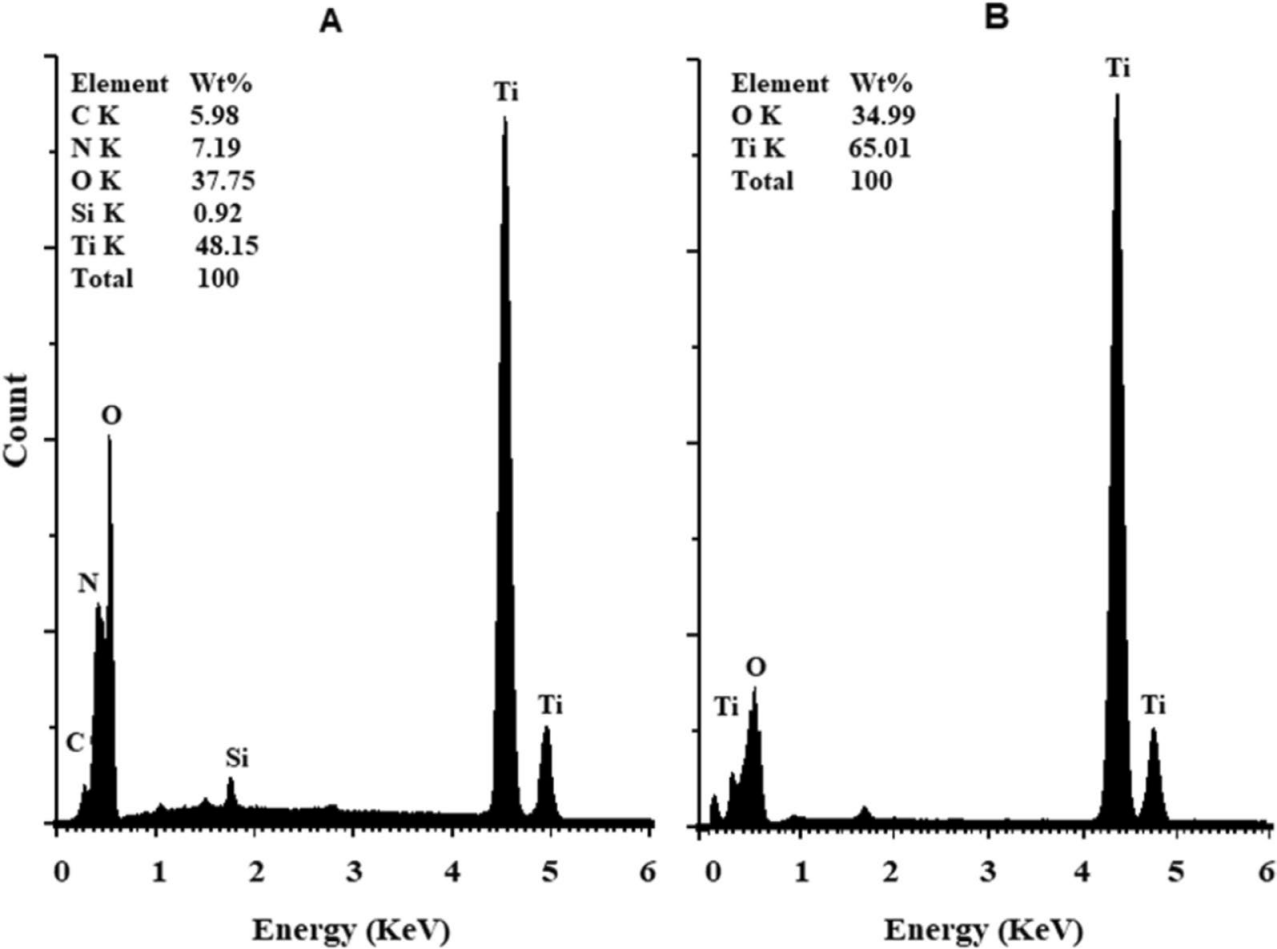
EDX spectra of uncalcined **A** and calcined **B** samples of biosynthesized TiO_2_ NPs

*Energy dispersive X-ray (EDX)* of TiO_2_ NPs before and after calcination is shown in Fig. 9*.* The uncalcined TiO_2_ sample's major constituents were oxygen (O; 37.75%) and titanium (Ti; 48.15%), in addition to weaker peaks of carbon (C; 5.98%) and nitrogen (N; 7.19%) (Fig. 9A), while the calcined sample showed only oxygen (49.93%) and titanium (50.07%) (Fig. 9B).

### NDSSCs performance

**Fig. 10:**
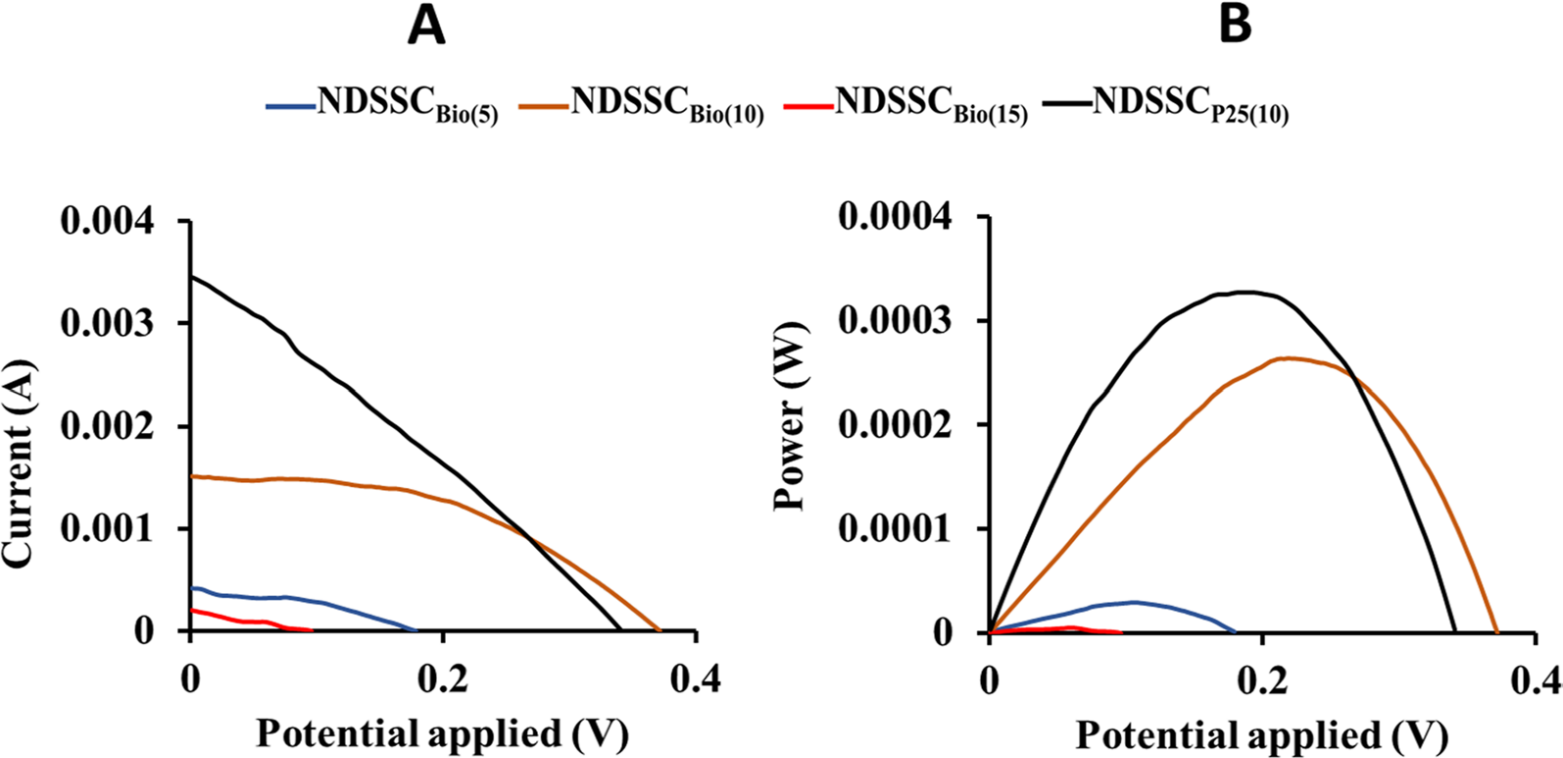
Photocurrent–voltage characteristics for NDSSC_Bio_ with different counter electrode cycles in comparison to NDSSC_P25(10)_. **A** *J-V* characterization. **B** P–V curve

The optimum number of SILAR cycles employed in the Cu_2_S counter electrode design in NDSSC_Bio_ was 10 cycles [NDSSC_Bio(10)_], with an efficiency (η) of 0.44%, an open-circuit voltage (V_OC_) of 213 mV, and a short-circuit current density (Isc) of 1.24E-03 mA/cm^2^, compared to an efficiency (η) of 0.55% resulting from NDSSC_P25(10)_ **(**Fig. 10**).** NDSSCs photovoltaic performance is summarized in Table 2.

**Table 2:** Photovoltaic parameters of the NDSSCs

DSSC	V_*OC*_ (V)	J*sc* (mA/cm^2^)	P_max_	FF	η (%)
NDSSC_Bio(5)_	1.06E-01 ± 0.0017	2.72E-04 ± 2.2E-06	2.88E-05 ± 7.8E-07	0.39 ± 0.045	0.048 ± 0.0036
NDSSC_Bio(10)_	2.13E-01 ± 0.0085	1.24E-03 ± 2.2E-05	2.64E-04 ± 1.1E-05	0.47 ± 0.022	**0.44** ± 0.0171
NDSSC_Bio(15)_	5.95E-02 ± 0.0088	8.92E-05 ± 2.1E-07	5.31E-06 ± 7.8E-08	0.27 ± 0.034	0.0088 ± 0.0002
NDSSC_P25(10)_	1.84E-01 ± 0.0116	1.78E-03 ± 1.3E-04	3.27E-04 ± 2.3E-05	0.28 ± 0.099	**0.55** ± 0.0236

The bold-style efficiency values represent the optimized NDSSCs

**Fig. 11:**
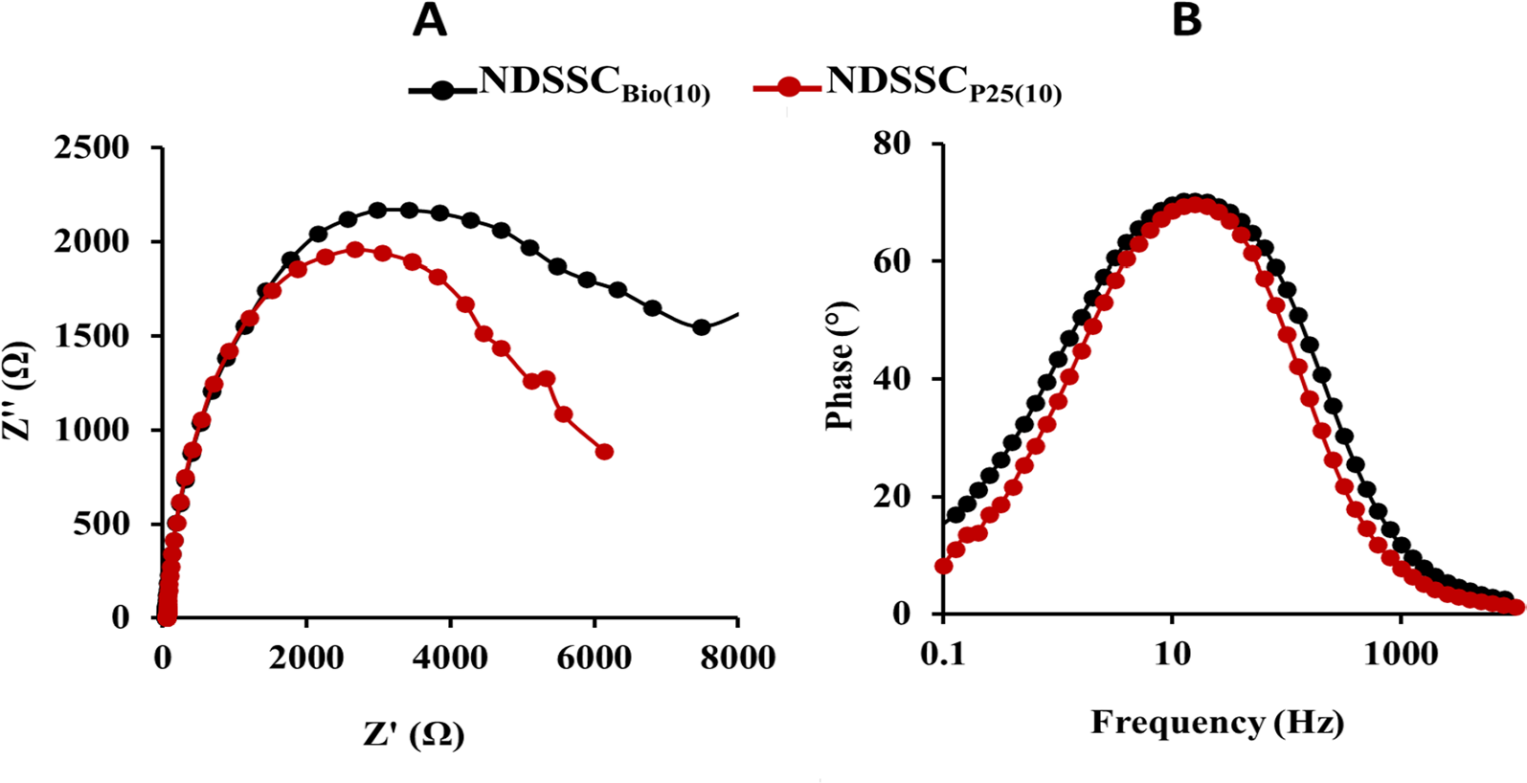
EIS of the optimized NDSSCs. Nyquist **A** and Bode **B** plots

EIS was recorded at frequencies ranging from 1 Hz to 10 kHz. It aims to analyze and characterize the major internal charge transfer resistances that limit the performance of the cells, which were recorded in the Nyquist (Fig. 11A) and Bode plots (Fig. 11B) of the optimized NDSSCs. Well-marked semicircles attributed to the charge transfer resistance between the Cu_2_S counter electrode and electrolyte were shown in the high-frequency regions. In the EIS analysis, a smaller diameter of Nyquist plots for the NDSSC_P25(10)_ indicated smaller charge transfer resistance (R_ct_) than that of the NDSSC_Bio(10)_, which explained its higher efficiency.

## Discussion

Some microorganisms can grow in conditions that most other organisms cannot. Halophiles are one of the most important microbial communities that can tolerate high salt concentrations and are highly sought after by a variety of industries for their unique enzymes and products with broader potential applications [35]. Members of the *Halomonadaceae* family can survive in moderate and Antarctic saline lakes, saline soils, and marine environments regardless of their geographical location. Our work led us to conclude that the starter culture of the *Halomonas* sp. RAM2 strain for TiO_2_ NPs synthesis was most preferably performed in a nutrient broth medium supplemented with 5% NaCl, pH = 8, grown at a temperature of 30 °C.

Many microorganisms are capable of producing nanoparticles via either intracellular or extracellular pathways. The current study deals with extracellular TiO_2_ NPs synthesis. In terms of application, this has a significant advantage over an intracellular synthesis process because it avoids additional processing steps needed to liberate the nanoparticles from the bacterial cell, either by sonication or by reaction with a suitable detergent [36]. Some studies have shown that TiO_2_ NPs with varying crystal sizes were synthesized extracellularly by bacteria such as *Aeromonas hydrophila* (40.5 nm) [37], *Bacillus amyloliquefaciens* (15.23–87.6 nm**) **[38], *Bacillus licheniformis* (16.3 nm) [39], *Bacillus subtilis* (66–77 nm) [40] and *Lactobacillus* sp.** (**24.63 nm**)** [41]. Microorganisms can modify the composition of the solution through the production of extracellular proteins, enzymes, organic polymers and secondary metabolites in the culture supernatant, which can influence formation by promoting or hindering the stabilization of the first mineral seeds. So, the metal ions are reduced to nanoparticles [37, 38, 42, 43, 69]. This explains the presence of C and N in EDX analysis even after several washing steps before calcination at 500 °C.

Controlling particle size is critical in nanoparticle synthesis. In the current study, optimization of reaction conditions for TiO_2_ NPs synthesis has been studied through RSM. One of the most important synthesis parameters was that of the filtrate pH. Some studies proved that pH variation affected the average particle size of TiO_2_ NPs, as the lower the pH, the smaller the particle size [44], which indicates that the pH is clearly related to TiO_2_ NPs stability. Because each type of NP is stable near the isoelectric point, a change in pH can affect the double-layer properties, boosting the probability of flocculation or coagulation [45, 46]. Previous studies proved that a smaller average size of TiO_2_ NPs (14 nm) was obtained at pH = 1 in comparison to 19 and 20 nm at pH 7 and 10, respectively [47]. In another study, the average crystallite size varied from 9.92 nm (pH 6.8) to 21.02 nm (pH 5), with the crystallite size decreasing to 7.77 nm in a highly acidic medium (pH 3.2) [48].

From the obtained TEM micrographs and XRD patterns, the conversion of biogenic TiO_2_ NPs from anatase to rutile phase began at 700 °C and ended at 800 °C, revealing the formation of a high-temperature stable anatase phase via the green method. This is attributed to the reconstructive action that involves the breaking and reforming of bonds [18]. Heat treatment distorts TiO_6_ octahedra during the phase transition. At 700 °C, lattice distortion and breaking of Ti–O bonds affect the removal of oxygen ions, defects, and new Ti–O bond formation. The oxygen vacancies may act as nucleation sites, facilitating the rutile phase formation. The Ti–O bonds are perfectly reconstructed at 800 °C, transition, TiO_2_ NPs crystal size increased, leading to a lower surface area [49, 50]. These findings point out that increasing the calcination temperature increases the crystallinity, size, and phase transformation of TiO_2_ NPs [51].

By studying the optical properties of the calcined TiO_2_ NPs, the band gap was determined. The indirect band gap of TiO_2_ decreases with increase of the calcination temperature from 500 °C to 700 °C which is in consistent with previous studies [52]. This might be due to the increase of the particle size and presence of a mixture of the two transition phases of anatase and rutile. The optical band gap of TiO_2_ NPs (3.2 eV) calcined at 500 °C was promoted as the most appropriate sample for working electrode fabrication of NDSSCs, as anatase TiO_2_ NPs have better photocatalytic activity than rutile TiO_2_ NPs in pure phases [53]. The complete conversion to the rutile phase at 800 °C resulted in a direct wider band gap which is attributed to crystal defects formed in the particles.

**Fig. 12:**
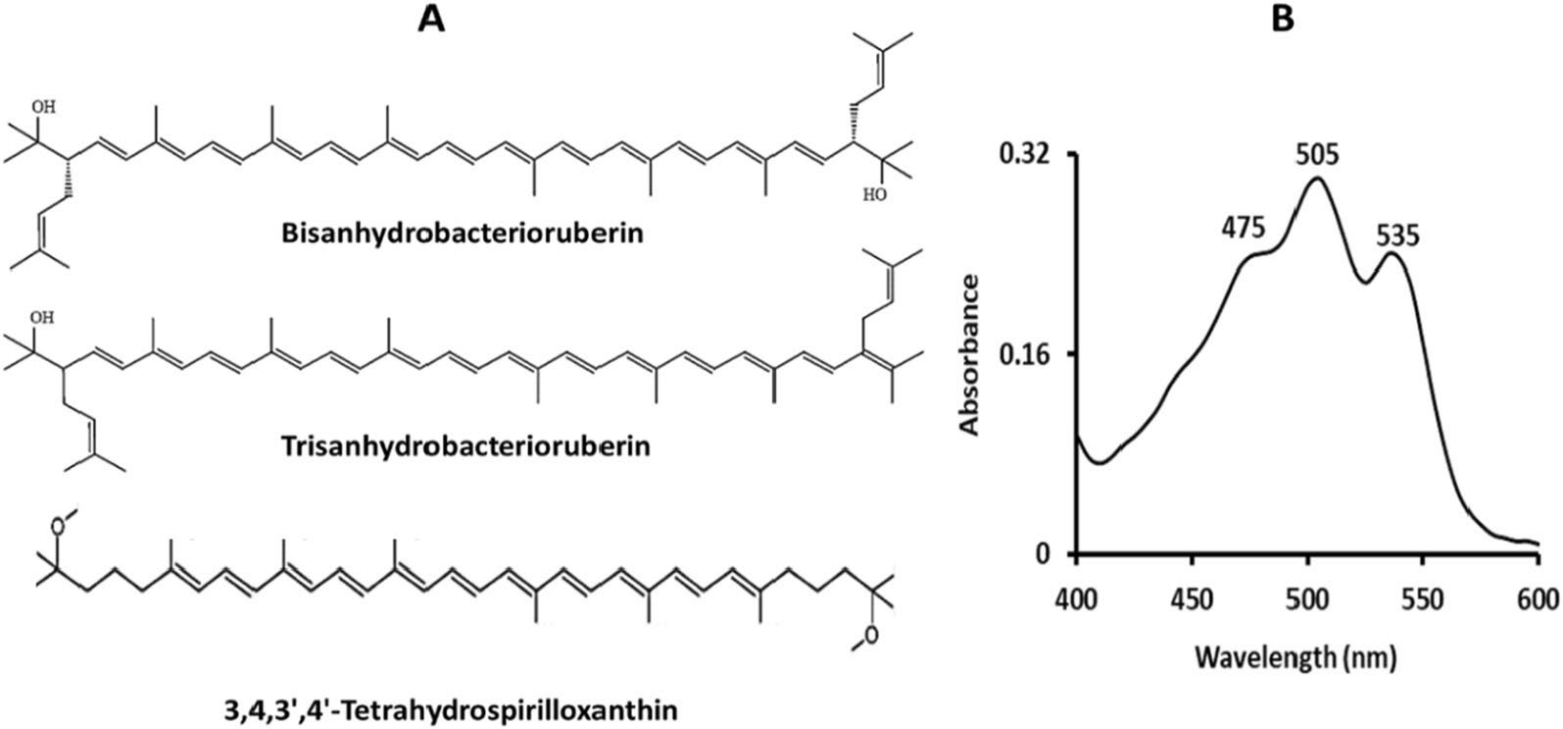
*Kocuria* sp. RAM1 carotenoids dye composition **A** and UV absorption **B**

Our NDSSCs consisted of TiO_2_ NPs film, carotenoids, redox polysulfide electrolyte, and a Cu_2_S counter electrode. Each component contributes to electron transport and diffusion. TiO_2_ acts as a scaffold for dye molecules that have been adsorbed and transports the electrons photogenerated by light absorption and dye regeneration [54]. The interaction between the *Kocuria* sp. RAM1 carotenoids and the TiO_2_ NPs resulted from the de-protonation of (-OH) groups of the carotenoids **(**Fig. 12A**)**, such as bisanhydrobacterioruberin and trisanhydrobacterioruberin. Bacterioruberins are types of C_50_-carotenoids with a broad absorption range in the visible region **(**Fig. 12B**).** They harvest the solar light, leading to being in an excited state, and then inject the photo-excited electrons into the conduction band (CB) of TiO_2_ NPs. Because the CB of TiO_2_ is at a lower energy level than the lowest occupied molecular orbital (LUMO) of carotenoids, injecting photoelectrons from carotenoids into TiO_2_ is energetically advantageous. Through the external circuit, the electrons travel to the counter electrode. The oxidized carotenoids accept electrons from the electrolyte, regenerating the ground state [55]. Several studies have reported the utilization of natural dyes extracted specifically from plants in DSSC designs, such as those from pomegranate and berry fruits [56], henna (Lawsonia inermis) leaves, beetroot [57], and tropical fruits [58].

Because of its simplicity, low temperature, and uniform layer deposition, the SILAR technique outperforms other methods [59]. A cycle (one layer) of Cu_2_S counter electrode film is deposited by the reaction at the substrate surface after the alternative adsorption of Cu^2+^ and S^2−^ ions. The observed optimum NDSSC efficiency was obtained after 10 cycles related to the uniformity, stoichiometry and band gap of the formed Cu_2_S film. As previously stated, there is a limited supply of S^2−^ ions from the anionic bath as the number of cycles increases [60].

To the best of our knowledge, this is a novel study that describes the use of C_50_-carotenoids extracted from marine bacteria as a sensitizer in NDSSCs that involve photo-induced charge transfer into the working electrode of the biosynthesized TiO_2_ NPs.

## Conclusion

Our study highlighted the eco-friendly TiO_2_ biosynthesis by the newly isolated halophilic marine bacteria *Halomonas* sp. RAM2 (OM276856). Also, the study was extended through the application of TiO_2_ NPs in NDSSC fabrication using the carotenoids as a natural dye extracted from *Kocuria* sp. RAM1 (OL904955). From an environmental perspective, attempting to fabricate DSSCs using an alternative green method is worthwhile, but further study is required to improve the obtained overall conversion efficiency.

## Methods

### Isolation, culture medium and identification

Halophilic bacteria were isolated from *Echinodermata* invertebrates collected from Safaga, Red Sea, Egypt. The samples were cut aseptically in sterile seawater, homogenized, and then kept in sterile bottles. One milliliter of each prepared sample was transferred into 100 ml of sterile nutrient broth made with distilled water and supplemented with 2% NaCl (w/v) before being incubated at 30 °C for 24 h under shaking conditions (120 rpm) before being isolated on agar plates for 72 h [61]. The pH was adjusted to 7 ± 0.2 before sterilization. Following incubation, colonies were purified and preserved as stock cultures for subsequent studies. For molecular identification, 16S rDNA was amplified by polymerase chain reaction (PCR) [62]. The PCR products were sequenced [63], and the BLAST program was used to explore the similarity [64]. The phylogenetic tree was generated using the software MEGA (Version 11.0.10) [65, 66].

### NaCl, pH and temperature effects on *Halomonas* sp. RAM2 growth

Bacterial growth (inoculum = 1%) was measured at 120 rpm at various NaCl concentrations (0–25% w/v), pH (5–10) and temperature (20–40 °C). The optical density at a wavelength of 600 nm for 48 h was used as a quantitative indicator [67, 68].

### Biosynthesis of TiO_2_ NPs using *Halomonas* sp. RAM2

**Fig. 13:**
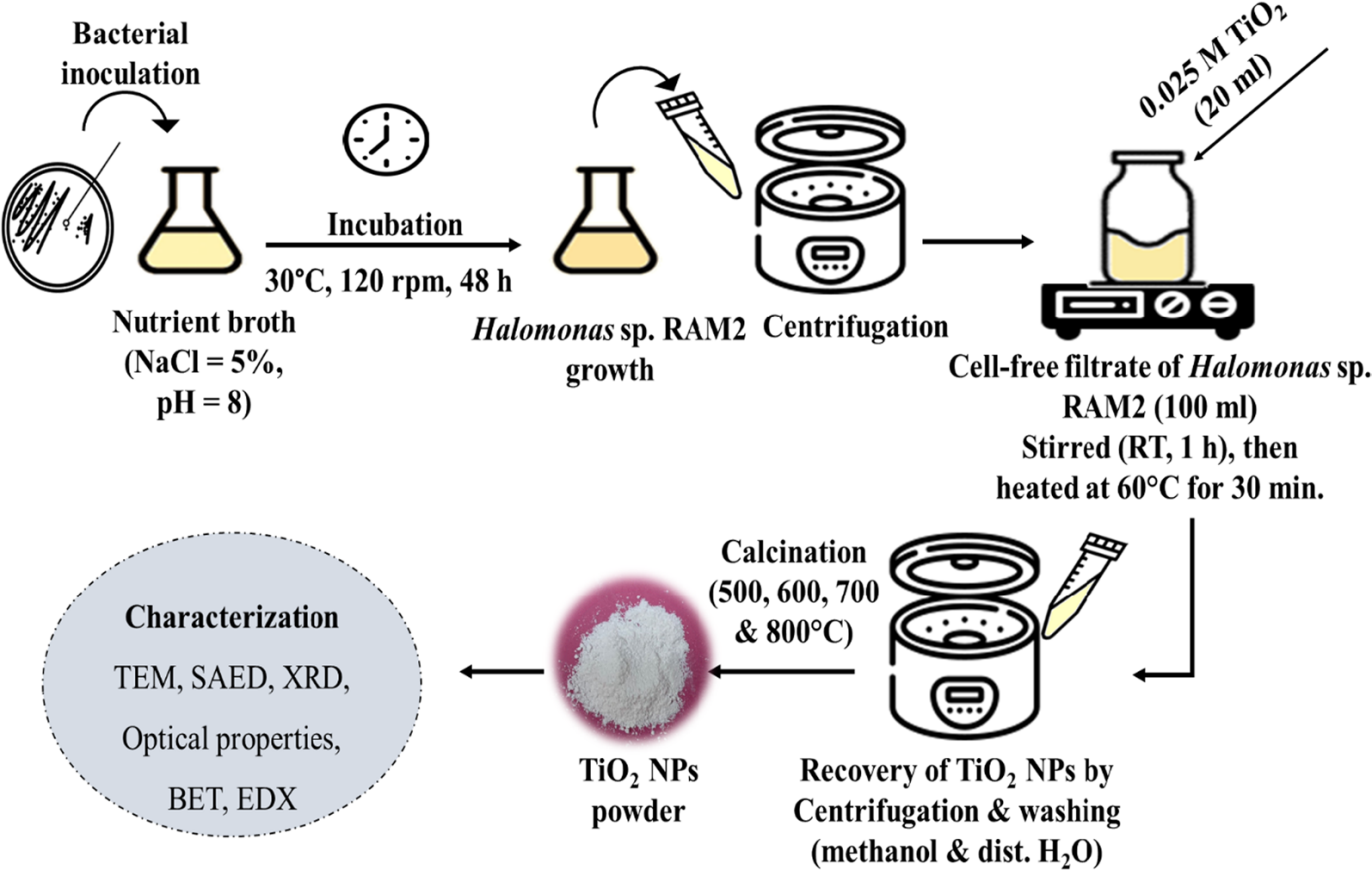
Schematic diagram of TiO_2_ NPs biosynthesis using *Halomonas* sp. RAM2

*Halomonas* sp. RAM2 seed culture was prepared under optimal conditions (NaCl = 5%, pH = 8, 30 °C) at 120 rpm for 48 h** (**Fig. 13**).** The culture supernatant was obtained after centrifugation of the broth at 6000 rpm for 15 min and filtrated. After that, the filtrate was challenged with 20 ml of 0.025 M TiO_2_, stirred at room temperature for 1 h, and then heated at 60 °C for 30 min. The biosynthesized TiO_2_ NPs were recovered by centrifugation, washed with methanol and distilled water several times, and then dried. The dried sample was calcined at 500, 600, 700, and 800 °C for 3 h for further studies [69].

### Optimization of reaction conditions on TiO_2_ NPs via response surface methodology (RSM)

The effect of reaction conditions on the TiO_2_ NP size via response surface methodology (RSM) through central composite inscribed (CCI) design was investigated using the cell-free filtrate of the *Halomonas* sp. RAM2 optimized growth [70]. Three independent variables using Design Expert (Version 11 Stat-Ease Inc., Minneapolis, MN, USA) were applied to investigate the effects of the starting TiO_2_ concentration (A), pH (B) of the cell-free filtrate, and the reaction duration (C) on TiO_2_ NPs size **(**Table 3**).** The following polynomial equation fits the experimental results:

2 $${\text{Y}} = \beta_{0} + \beta_{1} X_{1} + \beta_{2} X_{2} + \beta_{3} X_{3} + \beta_{12} X_{1} X_{2} + \beta_{13} X_{1} X_{3} + \beta_{23} X_{2} X_{3} + \beta_{11} X_{11}^{2} + \beta_{22} X_{22}^{2} + \beta_{33} X_{33}^{2}$$

where Y represents the response (TiO_2_ NPs size (nm)), *β*_*0*_ is constant, *β*_*1*_, *β*_*2*_, and *β*_*3*_ is linear coefficients, *β*_*12*_, *β*_*13*_, and *β*_*23*_ is cross product coefficients, *β*_*11*_*, β*_*22*_, and *β*_33_ is quadratic coefficients.

**Table 3:** Experimental independent variables and their coded levels for the central composite design

Name	Code	Levels of coded variables				
		− α	− 1	0	+ 1	+ α
TiO_2_ concentration (M)	A	0.005	0.0141214	0.0275	0.0408786	0.05
pH	B	5	5.8	7	8.2	9
Reaction duration (min)	C	60	72	90	107	120

The average size of TiO_2_ NPs was estimated using XRD analysis and the Scherrer’s formula. The model accuracy was determined by the coefficient of R^2^. The *P*-value for the significant model terms was set at 95%.

### Characterization of the biosynthesized TiO_2_ NPs

The shape, size and crystallinity of the biogenic TiO_2_ NPs were determined by TEM [71] (JEM-2100plus, JEOL, Japan)**,** SAED pattern [72], EDX spectrophotometer [73] and XRD [3]. XRD analysis was performed using an X-ray diffractometer with Cu-Kα crystal radiation (λ = 1.54060 Å) and scanning rate of (5°/min^−1^) and the scanning range of (10°—80°). The Scherrer equation was used to calculate the mean diameter of the NPs from the XRD pattern as follows [74]:

3 $${\text{D}}\, = \,\left( {0.9\,\lambda } \right)\,/\,\left( {\beta \,{\text{cos}}\theta } \right)$$

where λ = 1.5405 Å is the wavelength of the Cu-Kα radiation, and β is the full-width at half-maximum (FWHM) intensity in radians.

The TiO_2_ NPs optical properties were investigated using a UV/VIS spectrophotometer (Thermo Scientific) in the 200–900 nm wavelength range and the energy band gap was calculated using the Tauc plot (Eq. 4) [75]:

4 $$(ahv)^{(1/n)} = A(hv - E_{g} )$$

where α is the extinction coefficient, h is the Planck’s constant (J.S), *v* is the light frequency (s^−1^), A is the absorption constant, E_g_ is the energy band gap (eV), and n is the value of the specific transition (n = 2 for indirect band gap and n = 1/2 for direct band gap).

The Brunauer -Emmett and Teller (BET) (Quantachrome T ouchWin v1.2, USA) was used for determination of TiO_2_ NPs surface area after degassing the samples at 200 °C for 3 h [76], and Barret–Joyner–Halender (BJH) was used for pore size distribution determination [77].

### Fabrication of NDSSCs

Carotenoids as a photosensitizer were extracted from *Kocuria* sp. RAM1 as follows: One liter of a 48-h bacterial culture grown at 30 °C under shaking conditions was centrifuged for 15 min to collect the pellets. 250 ml of methanol were added to the pellet, which was then incubated in a water bath at 40 °C for 15 min until the dye recovered completely. The extract was purified, dissolved in petroleum ether, and stored in a refrigerator away from direct light [30].

**Fig. 14:**
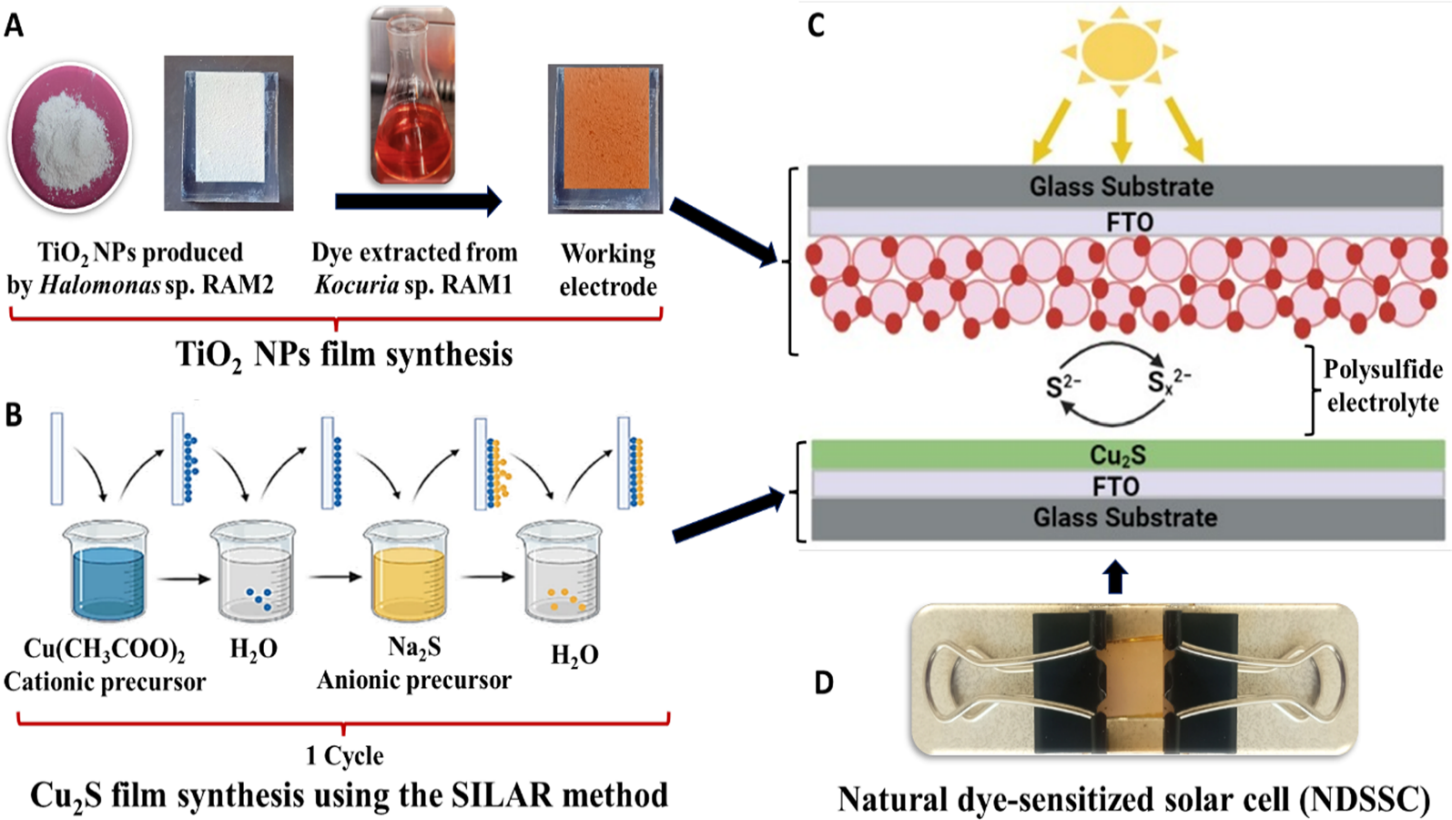
Illustration of NDSSC fabrication process. **A** Preparation of TiO_2_ NPs working electrode. **B** Preparation of Cu_2_S counter electrode via the SILAR method**. C** Scheme of NDSSC. **D** Fabricated NDSSC

A TiO_2_ working electrode was used in the fabricated DSSCs (Fig. 14A). The FTO substrate (15 Ω, Sigma) was cleaned with a detergent solution and successively sonicated for 30 min in soap, then distilled water, then acetone, and finally in isopropanol, respectively, followed by air drying. A TiO_2_ paste was made from a mixture of 0.5 g TiO_2_ NPs, 1.25 g α-terpineol, 0.25 g ethyl cellulose and a few drops of ethanol. The TiO_2_ homogeneous NPs paste was spread over the FTO substrate via a doctor blade coating technique, heated at 450 °C for 30 min, before being immersed in a concentrated carotenoids dye for 24 h at room temperature [78]. A polysulfide electrolyte solution was prepared from a mixture of 0.5 M Na_2_S, 0.1 M S, and 0.05 M KCl in ethanol: water (4:1 vol%) [20].

A Cu_2_S counter electrode films were fabricated on an FTO substrate using successive ionic layer adsorption and reaction (SILAR) technique [79]. The cationic precursor was a 0.5 M aqueous solution of copper acetate [Cu (CH_3_COO)_2_], while the anionic precursor was 0.5 M of sodium sulfide [Na_2_S]. A well-clean FTO-coated glass was immersed in copper acetate for 60 s. to promote ion adsorption on the surface of the substrate, before being immersed in double-distilled water for 20 s. to remove unadsorbed ions. The substrate was then immersed in sodium sulfide for 60 s before the last rinsing step in double-distilled water for 20 s. Thus, one deposition cycle was completed before being annealed in a furnace at 300 °C for 5 min (Fig. 14B). For optimization, the samples named NDSSC_Bio(5)_, NDSSC_Bio(10)_ and NDSSC_Bio(15)_ were prepared by repeating SILAR cycles 5, 10 and 15 times, and the most efficient system was compared to NDSSC equipped with TiO_2_ P25 (NDSSC_P25_) under the same conditions.

The NDSSC was assembled using carotenoid-sensitized TiO_2_ coated film that represents the working electrode, Cu_2_S film as a counter electrode and the polysulfide electrolyte solution was filled into the cells as illustrated in (Fig. 14C, D).

### NDSSCs characterization

The photovoltaic performance [short circuit current (Jsc), open circuit voltage (Voc), fill factor (FF), and power conversion efficiency (η)] of the fabricated DSSCs were measured under one sun (AM1.5G, 100 mW/cm^2^) illumination using a solar simulator. Electrochemical impedance spectroscopy (EIS) was evaluated using a computer-controlled potentiostat (NOVA 2.0**,** Metrohm Autolab) under dark conditions [16].

## Data Availability

All data generated or analyzed during this study are included in this published article. The datasets of the DNA sequence of the isolated bacterial strains (*Kocuria* sp. RAM1 and *Halomonas* sp. RAM2) analyzed during the current study are available in the GenBank repository under accession numbers OL904955 (https://www.ncbi.nlm.nih.gov/nuccore/OL904955) and OM276856 (https://www.ncbi.nlm.nih.gov/nuccore/OM276856.1/), respectively.
